# Developing a Dietary Lifestyle Ontology to Improve the Interoperability of Dietary Data: Proof-of-Concept Study

**DOI:** 10.2196/34962

**Published:** 2022-04-21

**Authors:** Hyeoneui Kim, Jinsun Jung, Jisung Choi

**Affiliations:** 1 The Research Institute of Nursing Science College of Nursing Seoul National University Seoul Republic of Korea; 2 Samsung Medical Center Seoul Republic of Korea

**Keywords:** dietary lifestyle data, person-generated health data, ontology, common data element, data interoperability, data standardization, dietary, health informatics

## Abstract

**Background:**

Dietary habits offer crucial information on one's health and form a considerable part of the patient-generated health data. Dietary data are collected through various channels and formats; thus, interoperability is a significant challenge to reusing this type of data. The vast scope of dietary concepts and the colloquial expression style add difficulty to standardizing the data. The interoperability issues of dietary data can be addressed through Common Data Elements with metadata annotation to some extent. However, making culture-specific dietary habits and questionnaire-based dietary assessment data interoperable still requires substantial efforts.

**Objective:**

The main goal of this study was to address the interoperability challenge of questionnaire-based dietary data from different cultural backgrounds by combining ontological curation and metadata annotation of dietary concepts. Specifically, this study aimed to develop a Dietary Lifestyle Ontology (DILON) and demonstrate the improved interoperability of questionnaire-based dietary data by annotating its main semantics with DILON.

**Methods:**

By analyzing 1158 dietary assessment data elements (367 in Korean and 791 in English), 515 dietary concepts were extracted and used to construct DILON. To demonstrate the utility of DILON in addressing the interoperability challenges of questionnaire-based multicultural dietary data, we developed 10 competency questions that asked to identify data elements sharing the same dietary topics and assessment properties. We instantiated 68 data elements on dietary habits selected from Korean and English questionnaires and annotated them with DILON to answer the competency questions. We translated the competency questions into Semantic Query-Enhanced Web Rule Language and reviewed the query results for accuracy.

**Results:**

DILON was built with 262 concept classes and validated with ontology validation tools. A small overlap (72 concepts) in the concepts extracted from the questionnaires in 2 languages indicates that we need to pay closer attention to representing culture-specific dietary concepts. The Semantic Query-Enhanced Web Rule Language queries reflecting the 10 competency questions yielded correct results.

**Conclusions:**

Ensuring the interoperability of dietary lifestyle data is a demanding task due to its vast scope and variations in expression. This study demonstrated that we could improve the interoperability of dietary data generated in different cultural contexts and expressed in various styles by annotating their core semantics with DILON.

## Introduction

### Background

Health-related lifestyle data (health lifelog data) provide essential information on one's daily behaviors that directly influence health; thus, such data needs to be used for the management of one's health [1-3]. Health-related lifestyle data are usually captured outside the clinical environment by patients and form a considerable part of the patient-generated health data (PGHD) [4]. Analyzing health-related lifestyle data with clinical, biological, and environmental data may offer additional insights into one's health status [5]. Despite its ever-increasing importance, challenges in data accuracy, data interoperability, and clinical workflow integration have been hindering the utilization of PGHD [6-8].

Capturing health lifelog data in a consistent and interoperable manner is a well-known challenge [9]. Similar to other types of PGHD, data interoperability is a significant challenge to reusing dietary data due to the inconsistent format and the colloquial terms used in describing the data. In addition, dietary data are captured through multiple channels with varying resolutions. One may directly type in the names and amounts of the foods she eats into a food diary app [10,11] or let an artificial intelligence (AI) camera app automatically recognize the type, amount, and nutritional components of the food [10]. Dietary habits are also often assessed using a questionnaire in many clinical or research settings. In this case, respondents need to recall their usual dietary habits for the past week, month, or even year.

The purpose of this study was to build an ontology for dietary behavior to address the interoperability challenge of dietary lifelog data. In particular, this study aimed to construct a dietary lifestyle ontology that incorporates culture-specific dietary concepts and demonstrates its utility in recognizing the data elements designed to assess similar dietary behaviors.

### Interoperability Challenges of Dietary Behavior Data

Lack of interoperability is one of the main barriers to using PGHD for patient care [12-14]. PGHD encompasses various health domains and is generated via multiple channels. For example, behavioral health history is collected using health questionnaires, while heart rate, physical activity, and sleep data are automatically captured through wearable sensor devices [15]. Dietary data are collected mainly by direct input from the users, either using a food diary app [16] or a dietary assessment questionnaire [17]. An AI camera automatically captures the type, amount, and nutritional components of the food that one eats, although users still need to confirm the accuracy of the captured information [10]. We may assess eating behavior at each moment of food intake or for a specific period as a health history. Because dietary data are presented in various forms and granularities, standardizing the data is not a trivial task.

Because diet-related lifestyle data are described mainly with nonprofessional terms and a free-text format, standardizing the data becomes more challenging. Dietary questionnaires usually adopt everyday language so that the respondents can easily understand the questions and provide accurate answers. For example, usual protein intake is assessed by asking how often people eat protein-rich food (eg, meat, milk, egg, tofu) in a given period instead of directly asking how many servings of protein they usually have. Therefore, numerous questions can be designed to obtain the same information using different food examples. This approach becomes a barrier to the interoperability of the questionnaire-based data in the nutritional domain unless the food examples are recognized as pointing to the same nutritional components.

Table 1 shows the questions designed to assess the same dietary behavior but with different food examples in different styles. English translations of the two examples from Korean dietary assessment scales are also provided in the table. Systematically identifying and recognizing these assessment items as similar or distinctive is an essential precursor to reusing these data for further analyses.

**Table 1:** Data elements sharing similar assessment topics.

Questions	Response options	Source	Logical Observation Identifiers Names and Codes (LOINC; version 2.72)
우유나 유제품(치즈, 요플레)을 얼마나 드십니까? [English translation: How often do you have milk or milk product (eg, yogurt, cheese)?]	거의 매일(주6-7일)^가끔(주3-5일)^거의 먹지않는다(주0-2일) [English translation: Almost every day (6-7 times per week), occasionally (3-5 times per week), or rarely (0-2 times per week)]	식습관 평가하기 (대한 영양사협회) [English translation: Diet checklist (the Korean Dietetic Association)]	N/A^a^
우유나 유제품(요구르트, 치즈) 등을 매일 먹는다. [English translation: I have milk or milk product (eg, yogurt, cheese) every day.]	예^가끔^아니오 [English translation: yes, occasionally, or no]	나의 식생활 평가표 (국가암정보센터) [English translation: My Diet Checklist (National Cancer Information Center)]	N/A
How often do you eat cheese (including on salads or in sandwiches or subs)?	Never or less than one time per month, one time per month, 2-3 times per month, 1-2 times per week, 3-4 times per week, 5-6 times per week, or one or more times per day	Consensus measures for phenotypes and exposures	61441-2
How often do you eat yogurt?	Never or less than one time per month, one time per month, 2-3 times per month, 1-2 times per week, 3-4 times per week, 5-6 times per week, or one or more times per day	Consensus measures for phenotypes and exposures	61457-8
Each time you eat cheese, how much do you usually eat?	less than one slice, one slice, or more than one slice	Consensus measures for Phenotypes and Exposures	61442-0

^a^N/A: not applicable.

### Approaches to Standardizing Dietary Behavior Data

#### Common Data Elements

Common Data Element (CDE) is the most popular standardization approach to health-related lifestyle data, especially patient-generated health history data. The National Institutes of Health (NIH) maintains the CDE repository containing various structured data elements that health researchers should consider adopting to produce interoperable data [18]. The CDE repository provides detailed information on a data element, including definition, the development context, and standardized concept mapping with Logical Observation Identifiers Names and Codes if available [18]. The well-organized and reusable CDEs certainly help us address the interoperability challenges of questionnaire-generated data to some extent. However, treating an entire data element as a single concept makes a data element distinctive if it contains a slight variation in the wording of a question or a response option, as shown in Table 1.

#### Metadata Annotation

**Figure 1:**
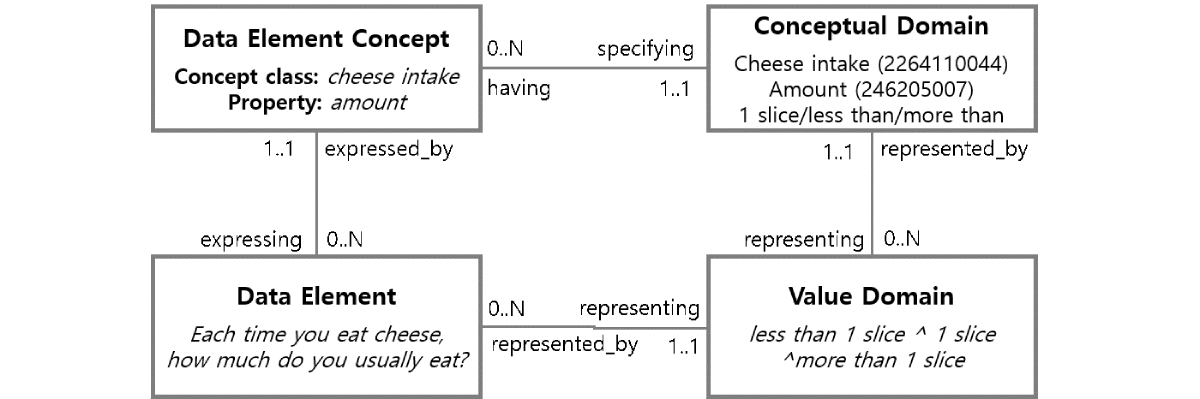
The International Organization for Standardization/International Electrotechnical Commission 11179 metadata model (Systematized Nomenclature of Medicine concept ID is included in parentheses).

The International Organization for Standardization/International Electrotechnical Commission (ISO/IEC) 11179 metadata registry (MDR) standard offers a means of attaching more detailed semantic information to data elements [19]. MDR standard-based metadata annotation requires specifying concept layers and representation layers of a data element (Figure 1). Data element concepts are divided into object classes and properties in a concept layer. The data element concepts and the response options (ie, value domain) are coded with standardized terminologies [19]. The concept layer of a data element enables us to identify the data elements that share similar assessment topics and permissible values, which thus can be analyzed together. However, existing standardized biomedical terminology systems provide limited dietary lifestyle concepts (and other health-related lifestyle concepts); therefore, it is hard to achieve complete vocabulary encoding of the concepts identified in the conceptual domain annotation.

Systematized Nomenclature of Medicine (SNOMED) is a comprehensive health terminology containing many health-related lifestyle concepts [20]. Even if appropriate concept codes are available in SNOMED [20], identifying similar or related questions through the conceptual structure of SNOMED is cumbersome because SNOMED is a large and complex system encompassing the broad scope of a health domain. The concepts relevant to dietary lifestyle are indeed scattered across the conceptual structure of SNOMED. In this regard, metadata annotation with the MDR standard alone is still limited in achieving interoperability of dietary lifestyle data, even though it enables capturing more detailed semantic information of a data element with standardized terminology encoding.

#### Ontologies for Food and Dietary Behavior

Multiple ontologies have been developed to serve various food and nutrition domains. A few examples include tracking the food production process [21], representing nutrient-related isotopic information [22], incorporating metabolomics of nutrients [23], systematically organizing culture-specific food names [24], recommending food for global travelers [25], and representing food additives for food safety [26]. Finally, FoodOn is a comprehensive food ontology that aims to be “a farm to fork ontology,” encompassing the concepts from food production to the food names in a restaurant menu [27].

Several studies reported adopting a food ontology to support various health management tasks. For example, food recommendation systems that targeted patients with specific chronic medical conditions were built based on food ontologies [28,29]. Applications that monitor one's general behavioral health and provide personalized healthy lifestyle choices were developed based on an ontology covering a dietary domain [30,31]. These studies indicated that ontologies had been actively used to represent dietary concepts in health domains. However, the ontologies used in these efforts primarily focused on describing food names, nutritional components, and the food production process. The existing ontologies have not sufficiently covered the concepts of diet-related lifestyle.

### Food Is a Cultural Concept

As a dietary lifestyle, we are primarily concerned about the types, amount, and time of food intake. A dietary lifestyle depends highly on one's culture. Food names, cooking methods, and food preservation methods vary across different regions and populations. Therefore, the scope and the number of concepts of dietary behavior are inherently vast, and developing a single standardized vocabulary system that encompasses all dietary concepts in the world is a daunting task.

FoodOn is a highly sophisticated and comprehensive food ontology covering food sources, categories, products, and manufacturing concepts [27,32]. However, it misses many culture-specific dietary concepts. For example, FoodOn lacks iconic food or common dietary behaviors frequently used to assess Korean people's eating habits. Typical unhealthy dietary patterns in Korea include eating convenience store food, late-night delivery food, and too much hot and spicy instant noodle soups (ramen). Examples of salty food are Jeot-gal and Jang-a-chi, crudely translated in English as salted fish and pickled vegetables. Various seasoned vegetables (namul-muchim) and kimchi are typically considered healthy food in Korean culture. These typical Korean foods and dietary patterns are not included in the food ontology developed in Western countries, including the most comprehensive one like FoodOn. A few culture-specific diet ontologies exist, but their primary developmental goals were to demonstrate modeling domain knowledge with an ontology [33] or organizing specific food names and nutritional components [24]. Therefore, they were not extensible to incorporate dietary lifestyle concepts from different cultural contexts.

### Purpose

The primary purpose of this study was to develop a dietary ontology with an extensible concept structure to support the interoperability of dietary lifestyle data from different cultural contexts. This study also demonstrated the utility of this ontology by addressing the interoperability issues presented in a set of ontology competency questions prepared for this study.

## Methods

### Developing Competency Questions

To guide the ontology development and evaluation process, we first prepared a set of ontology competency questions. The primary purpose of developing a dietary lifestyle ontology in this study is to support the interoperability of questionnaire-generated dietary behavior data by explicitly capturing the main concepts and relationships of a data element. Therefore, we designed the competency questions reflecting the use cases of systematically identifying data elements assessing similar dietary behaviors based on assessment topics or measurement types (Textbox 1).

**Competency questions.**

**CQ1**. Identify the questions by the assessment domain.

- **CQ1.1** questions on protein intakes.
- **CQ1.2** questions on carbohydrate intakes.
- **CQ1.3** questions on unhealthy eating behaviors.

**CQ2**. Identify the questions by assessment properties.

- **CQ2.1** assessing frequencies.
- **CQ2.2** assessing general degrees.
- **CQ2.3** assessing applicability (ie, true or false).

**CQ3**. Identify the questions that specify an observation period.

- **CQ3.1** assessing the behavior of the past month.
- **CQ3.2** assessing the behavior of the past 6 months.

**CQ4**. Identify the questions that share the same assessment domain and property.

- **CQ4.1** questions on the frequency of high-calorie food intake.
- **CQ4.2** questions on the applicability of unhealthy eating behavior.

### Collecting Dietary Terms and Concepts From Existing Questionnaires

Diet-related data elements were retrieved from the NIH's CDE repository with “eating,” “diet,” and “nutrition” as search keywords. Korean dietary assessment questionnaires were retrieved from a web search using similar search keywords. These searches yielded 367 unique Korean data elements and 791 unique English ones. Note that the data elements were treated as distinctive unless expressed in precisely the same words and styles, even if they carry the same information. For example, the examples presented in Table 1 were treated as distinctive data elements.

For this study, 2 nursing students and 1 medical informatics student reviewed and curated the questions following the ISO/IEC 11179 MDR standard [19]. This metadata curation includes identifying essential concepts such as extracting data element concepts from the questions and dividing the data element concepts into an object class and properties. The object class and properties were decomposed into atomic dietary concepts when necessary. This concept extraction process yielded 515 unique dietary concepts (224 from Korean data elements, 363 from English data elements, and 72 overlapping Korean and English data elements). The concepts extracted from the questions and the permissible values were the main ingredients for building the dietary lifestyle ontology.

### Reviewing Existing Food and Nutrition Ontologies

We reviewed the FoodOn [27] ontology to benchmark its high-level conceptual structure. Major concept branches (or axes), concept categorization schemes, and higher-level class labels were examined and borrowed to construct the backbone structure of the dietary lifestyle ontology. The food product and food transformation process classes of FoodOn provided valuable insights into categorizing food names and food handling methods.

### Ontology Building

The authors reviewed the extracted terms and grouped them into broad categories benchmarking the existing ontologies. The broad categories were food items, food preparation or handling, eating behaviors, and descriptors. These top-level categories were further divided into subcategories representing more specific dietary concepts.

The scope of the dietary domain is vast, spanning from specific food names to physical and mental health status related to food intake. In particular, incorporating the names of all the existing food in the world in this ontology is a demanding and maybe unnecessary task. Therefore, the concept branch that includes food names is structured to facilitate the future addition of food names. The food item class focuses on representing broad types of food, and specific food names were added as instances.

We first built a draft ontology that included every concept we extracted from the text source (ie, 367 Korean data elements and 791 English data elements). We then pruned the ontology by removing the concepts that are too peculiar (eg, salty snack) or already well reflected in other related concepts. For example, we removed “eating out” and kept “restaurant food,” “delivery food,” and “convenience store food.” We also added new concepts to make the concept classes more complete. For example, “appetizer” and “chopstick” were added to the meal course and the utensil classes, respectively, although our text source did not include those concepts.

**Figure 2:**
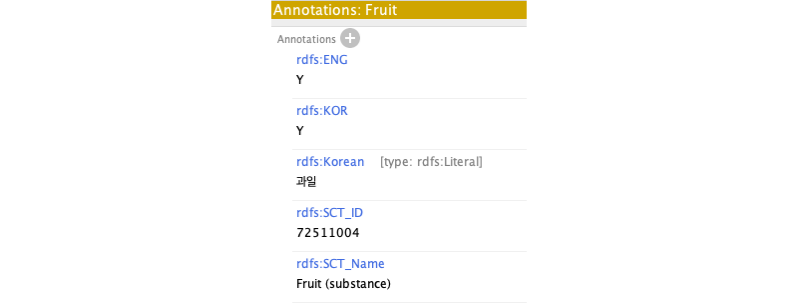
Annotation of a concept.

We built the dietary lifestyle ontology considering the structural component of the ontology quality evaluation and requirement (OQuaRE) [34]. A few examples of the structural elements of OQuaRE include cohesiveness, consistency, lack of redundancy, lack of cyclic structure, and good domain coverage [34]. The concept classes were labeled in English, even those extracted from a Korean questionnaire. All concepts took a singular noun form, and the first character of a concept label was capitalized. Each concept was annotated with a Korean translation of the concept, language of the concept source (ie, Korean questionnaire or English questionnaire), and SNOMED CT (SNOMED Clinical Terms) concept codes when available. We used the September 1, 2021, version of the US edition SNOMED CT (Figure 2).

### Ontology Evaluation

We evaluated the dietary lifestyle ontology built in this study for concept coverage, structural quality, and utility.

#### Concept Coverage

We evaluated the concept coverage of the dietary lifestyle ontology with the concepts extracted from the dietary habit questions not used for the ontology building. We pulled 374 concepts from 134 Korean data elements and 92 concepts from 50 English data elements using the same process described in the “Collecting dietary terms and concepts from existing questionnaires” of the methods section. We then examined the extent to which the dietary lifestyle ontology covered this newly prepared set of concepts.

#### Structural Quality

We evaluated the structural quality of the dietary lifestyle ontology using the ontology Debugger plug-in of Protégé [35] and OntOlogy Pitfall Scanner! (OOPS!) [36]. The Debugger plug-in of Protégé examines the consistency and the coherency of an ontology. If an ontology contains a class defined with two conflicting conditions (ie, unsatisfiable), the ontology is incoherent [35]. At the same time, an unsatisfiable class cannot have an instance that fits the class definition; thus, the ontology is inconsistent [35]. OOPS! is a web-based application that evaluates the quality of an ontology against common errors of ontology authoring based on state-of-the-art ontology authoring principles [37].

#### Utility

**Figure 3:**
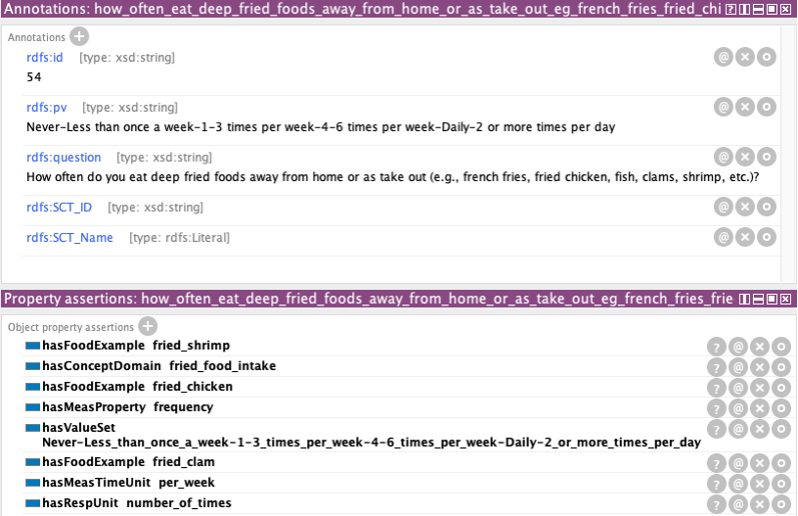
An example of the instantiated data elements.

We evaluated the utility of the dietary lifestyle ontology by finding answers to the competency questions (CQs) presented previously. To answer the CQs, we randomly selected 68 dietary assessment data elements that we had analyzed to build this ontology and instantiated them under the data element class. The data element class has two subclasses of question and value set. We instantiated the questions under the question class and their permissible values under the value set class. The question instances took an abbreviated form for simplicity. The complete data element information, such as a full question sentence and the associated permissible value set, was captured as an annotation for better legibility. Figure 3 shows how a data element instance, “how often eat deep-fried foods away from home or take out,” is annotated and defined with object properties for the utility testing.

Table 2 presents the object properties constructed to capture a data element's semantics and bind its value set to its question.

We developed query rules with the Semantic Query-Enhanced Web Rule Language (SQWRL) rules to retrieve the data element instances that satisfy the conditions of the CQs. The authors reviewed the query results for accuracy.

**Table 2:** The object properties.

Object property name	Purpose	Example
hasConceptDomain	The core topic area of a data element	Fast food intake, dairy food intake, eating speed, etc
hasMeasProperty	The property of the topic area	Frequency, amount, etc
hasFoodExample	Food examples used to explain the core topic area	Yogurt, macaroni, cheese, fruit juice, etc
hasMeasTimeUnit	Unit of assessment duration	Per day, per month, per week
hasObsPeriod	Observation period that a data element represents	Past 3 months, past week, past 6 months, etc
hasRespUnit	Response unit	Number of days, number of times, calories, etc
hasValueSet	A set of permissible values	Less than one cup, one cup (8 ounces), more than one cup

## Results

### The Dietary Lifestyle Ontology

**Figure 4:**
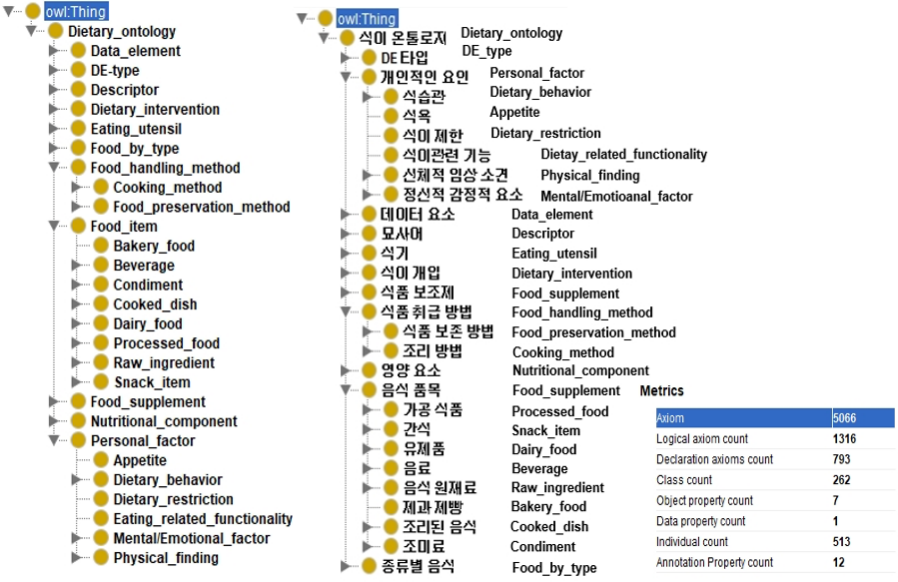
The top-level hierarchy and key metrics of the dietary lifestyle ontology.

The Dietary Lifestyle Ontology (DILON) was built with 262 concept classes, 513 instances, and 7 object properties. The final version of the ontology contained 224 concepts from Korean questionnaires, 363 concepts from English questionnaires, and 233 added concepts. Only 72 concepts appeared in both the Korean questionnaires and the English questionnaires. Figure 4 shows the top-level hierarchy and the key metrics of this ontology. The concept hierarchy rendered in Korean is offered with an English translation of each class label.

### Ontology Evaluation Results

The test of concept coverage showed that DILON covered most of the dietary concepts from the text source prepared for the ontology evaluation. Only 19 concepts from Korean questions and 6 from English questions were not covered in DILON. A few examples of the concepts missing in DILON were green dietary life, food combination, retching, and teaspoon.

The Debugger plug-in for Protégé confirmed that DILON has a consistent and coherent structure. OOPS! suspected that some equivalent concepts were defined as distinctive in DILON. A few examples of the concept pairs that OOPS! suspected as equivalents were regurgitation and vomiting, frying and sauteing, grain and cereal, shellfish and mollusk, and thirst and hunger. Because these concept pairs are not equivalent, we concluded that OOPS! did not find any substantial pitfalls in DILON. DILON is available in the National Center for Biomedical Ontology BioPortal [38].

**Figure 5:**
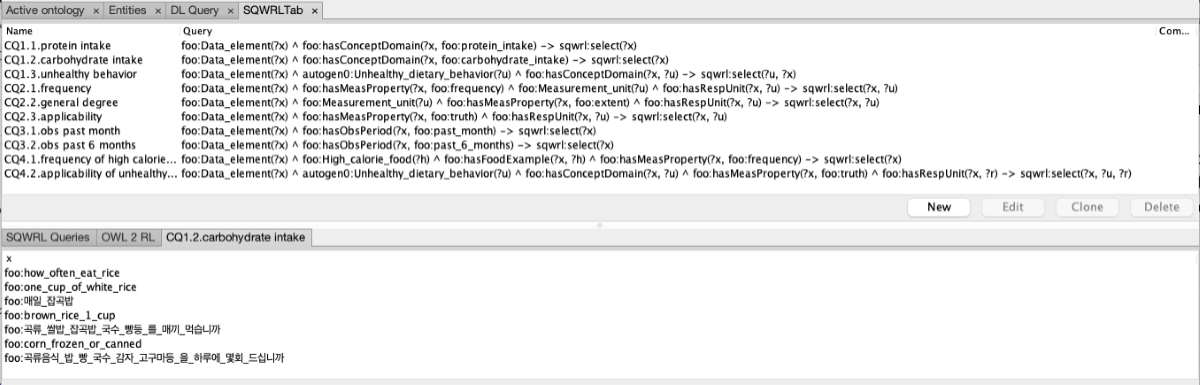
CQ evaluation using SQWRL. CQ: competency question; SQWRL: Semantic Query-Enhanced Web Rule Language.

The SQWRL [39] implementation of the competency questions is presented in Figure 5. The SQWRL queries correctly identified the data elements that meet the conditions specified in the CQs. The SQWRL query results demonstrated that it is possible to systematically identify the data elements that share similar assessment topics, measurement methods, and response types when the main concepts of the data elements are annotated with DILON. The full results and SQWRL queries are presented in the supplementary file.

## Discussion

### Principal Findings

This study demonstrated improving the interoperability of questionnaire-generated dietary lifestyle data using the proposed ontology DILON. Dietary concepts and their relationships in DILON are useful for resolving the challenges introduced when treating an entire diet-related data element as a single concept. Explicitly capturing the core semantics of a data element with the concepts and the object properties of DILON enabled us to identify the data elements that carry similar information. It is an important step toward achieving interoperability of questionnaire-generated data that can have many variations of expression for the same information.

Eating food is a cultural concept. Thus, diet-related concepts such as food names and dietary patterns vary by culture. We observed clear distinctions in food examples between Korean questionnaires and English questionnaires. FoodOn is an extensive and highly comprehensive system. Although FoodOn contains the Cultural Food Product class, this class includes only twelve food examples as subclasses. The Prepared Food Product class of FoodOn contains a long list of cooked dish names. Some of those cooked dish names are generic (eg, leafy vegetable, potato soup, sandwich), and some have a specific cultural origin (eg, brochette, chow mein, tataki). The Prepared Food Product class probably does not intend to be a complete list of culture-specific food names; thus, most typical food names from specific cultures are missing. Consistent and unambiguous representation of various food names and their nutritional information is crucial for analyzing and interpreting dietary behavior data. Of note, only 54% of the dietary concepts in DILON were mapped to SNOMED CT.

Developing a single comprehensive dietary ontology covering all food and dietary behavior concepts is a daunting task. Instead, a more feasible approach is creating a smaller yet extensible ontology representing the dietary concepts required for a specific task or a given cultural context. In addition, separating the portions common to any cultural contexts from those culture-specific would make an ontology easily extensible and later integrable with other specialized food ontologies when the need arises. For that reason, in DILON, dietary concepts common to any cultural context were created as classes with a relatively simple hierarchy, and food names were included as instances. DILON does not intend to be a comprehensive culinary taxonomy. Therefore, complex hierarchical information among food types is omitted in DILON to keep the concept structure manageable.

Many apps offer efficient ways to capture dietary intake patterns and produce additional information such as nutritional components and calorie intake. Nonetheless, questionnaire-based assessment is still helpful for capturing general patterns of dietary behavior and diet-related health history. The dietary ontology like DILON may also facilitate the integration of app-generated data and questionnaire-generated data.

### Limitations

This study has several limitations. The first is using the relatively limited text sources to identify dietary lifestyle concepts. DILON was built with the terms and concepts collected from existing food ontologies and a limited number of dietary assessment questionnaires. Therefore, the current version of DILON is a small system with relatively limited content coverage, and continuous augmentation is warranted. The second is methodological scalability. DILON was developed through a manual process. Thus, the methods adopted in this study may not be scalable. We need to establish an algorithmic strategy to facilitate continuous update and augmentation of DILON. The third limitation is the scope of the utility testing. The utility testing of DILON depended on the competency questions that reflect a narrow spectrum of interoperability challenges. Therefore, the utility of DILON in addressing interoperability challenges of dietary data needs to be demonstrated through a real data integration use case in a future study.

### Conclusions

Dietary lifestyle data offer crucial information on one's health, but the interoperability challenges are a significant barrier to reusing the data. The vast scope of dietary concepts, the colloquial style of expression, and the various cultural contexts reflected in dietary lifestyle data make standardizing the data a demanding task. This study showed that we could improve the interoperability of the dietary lifestyle data generated from questionnaires in different cultural contexts by annotating essential semantic information of dietary data elements with the dietary lifestyle ontology DILON.

## Appendix

Multimedia Appendix 1 Semantic Query-Enhanced Web Rule Language result table.

## Abbreviations

AI: artificial intelligence
CDE: Common Data Element
CQ: competency question
DILON: Dietary Lifestyle Ontology
IEC: International Electrotechnical Commission
ISO: International Organization for Standardization
MDR: metadata registry
NIH: National Institutes of Health
OOPS!: OntOlogy Pitfall Scanner!
OQuaRE: Ontology Quality Evaluation and Requirement
PGHD: patient-generated health data
SNOMED: Systematized Nomenclature of Medicine
SNOMED CT: Systematized Nomenclature of Medicine Clinical Terms
SQWRL: Semantic Query-Enhanced Web Rule Language
