# Supplementary material for: Developing a Dietary Lifestyle Ontology to Improve the Interoperability of Dietary Data: Proof-of-Concept Study
Source: JMIR Form Res. 2022 Apr 21;6(4):e34962. doi: 10.2196/34962 (PMC9073603; doi:10.2196/34962)
Supplement: Multimedia Appendix 1 [file formative_v6i4e34962_app1.docx]

| **Competency Question 1. questions on protein intake** | | | | | |
| --- | --- | --- | --- | --- | --- |
| SQWRL syntax | foo:Data_element(?x) ^ foo:hasConceptDomain(?x, foo:protein_intake) -> sqwrl:select(?x) | | | | |
| x | foo:두부_쇠고기_생선등_단백질식품을_하루_1회이상_섭취합니다 | | | | |
|  | foo:하루_2끼_이상_고기_생선_달걀_콩_두부_섭취 | | | | |
|  | foo:생선_고기_계란_콩_두부등으로_만든_반찬을_하루에_몇회_드십니까 | | | | |
| **Competency Question 2. questions on carbohydrate intake** | | | | | |
| SQWRL syntax | foo:Data_element(?x) ^ foo:hasConceptDomain(?x, foo:carbohydrate_intake) -> sqwrl:select(?x) | | | | |
| x | foo:how_often_eat_rice | | | | |
|  | foo:one_cup_of_white_rice | | | | |
|  | foo:매일_잡곡밥 | | | | |
|  | foo:brown_rice_1_cup | | | | |
|  | foo:곡류_쌀밥_잡곡밥_국수_빵등_를_매끼_먹습니까 | | | | |
|  | foo:corn_frozen_or_canned | | | | |
|  | foo:곡류음식_밥_빵_국수_감자_고구마등_을_하루에_몇회_드십니까 | | | | |
| **Competency Question 3. questions on unhealthy eating behavior** | | | | | |
| SQWRL syntax | foo:Data_element(?x) ^ autogen0:Unhealthy_dietary_behavior(?u) ^ foo:hasConceptDomain(?x, ?u) -> sqwrl:select(?u, ?x) | | | | |
| u, x | foo:overeating | foo:eat_till_uncomfortably_full | | | |
|  | foo:fastfood_intake | foo:how_often_eat_pizza | | | |
|  | foo:binge_eating | foo:how_many_days_per_week_on_average_over_the_past_6_months_you_eat_an_usually_large_amount_of_food_and_experienced_a_loss_of_control | | | |
|  | foo:overeating | foo:과식을_합니까 | | | |
|  | foo:instant_food_intake | foo:햄_라면_햄버거_인스턴트_가공식품_섭취 | | | |
|  | foo:processed_food_intake | foo:햄_라면_햄버거_인스턴트_가공식품_섭취 | | | |
|  | foo:overeating | foo:과식하지_않는다 | | | |
|  | foo:overeating | foo:과식을_하는_경우가_있습니까 | | | |
|  | foo:binge_eating | foo:억제_할수없이_폭식한_적이_있다 | | | |
|  | foo:fastfood_intake | foo:each_time_you_eat_pizze_how_much_do_you_usually_eat | | | |
|  | foo:instant_food_intake | foo:육가공식품_햄_베이컨_소시지등이나_라면_인스턴트식품을_1주일에_3회이상_먹는다 | | | |
|  | foo:processed_meat_intake | foo:육가공식품_햄_베이컨_소시지등이나_라면_인스턴트식품을_1주일에_3회이상_먹는다 | | | |
|  | foo:processed_meat_intake | foo:how_often_eat_sausage_bacon_regular_fat | | | |
|  | foo:instant_food_intake | foo:패스트푸드_라면_과자등의_인스턴트_식품 | | | |
|  | foo:binge_eating | foo:음식에_대한_조절력을_상실하여_한꺼번에_많은양의_음식을_매우_빨리먹는다 | | | |
|  | foo:eating_fast | foo:음식에_대한_조절력을_상실하여_한꺼번에_많은양의_음식을_매우_빨리먹는다 | | | |
| **Competency Question 4. assessing frequency** | | | | | |
| SQWRL syntax | foo:Data_element(?x) ^ foo:hasMeasProperty(?x, foo:frequency) ^ foo:Measurement_unit(?u) ^ foo:hasRespUnit(?x, ?u) -> sqwrl:select(?x, ?u) | | | | |
| x, u | foo:how_many_time_per_week_on_average_over_the_past_3_months_you_eat_an_usually_large_amount_of_food_and_experienced_a_loss_of_control | | | | foo:number_of_days |
|  | foo:during_past_month_how_often_drink_100_fruit_juice_such_as_orange_mango_apple_and_grape_juices | | | | foo:number_of_times |
|  | foo:패스트푸드_라면_과자등의_인스턴트_식품 | | | | foo:number_of_days |
|  | foo:how_often_eat_sausage_bacon_regular_fat | | | | foo:number_of_times |
|  | foo:과일을_얼마나_드십니까 | | | | foo:number_of_days |
|  | foo:how_often_eat_rice | | | | foo:number_of_times |
|  | foo:당근_시금치등의_녹황색_채소를_먹습니다 | | | | foo:number_of_days |
|  | foo:how_often_eat_french_fries_home_fries_or_hash_brown_potatoes | | | | foo:number_of_times |
|  | foo:how_often_you_eat_cheese_including_on_salads_or_in_sandwich_or_subs | | | | foo:number_of_times |
|  | foo:how_often_eat_macaroni_and_cheese | | | | foo:number_of_times |
|  | foo:과일을_매일_섭취합니다 | | | | foo:number_of_days |
|  | foo:how_often_eat_egg_fried_scrambled_in_margarine_butter_oil | | | | foo:number_of_times |
|  | foo:과식을_하는_경우가_있습니까 | | | | foo:number_of_times |
|  | foo:how_often_eat_beef_pork_hotdogs_regular_fat | | | | foo:number_of_times |
|  | foo:how_often_eat_deep_fried_foods_away_from_home_or_as_take_out_eg_french_fries_fried_chicken_fish_calms_shrimp_etc | | | | foo:number_of_times |
|  | foo:during_past_month_how_often_eat_whole_grain_bread_including_toast_rolls_and_in_sandwiches | | | | foo:number_of_times |
|  | foo:fresh_apples_or_pears | | | | foo:number_of_times |
|  | foo:during_past_month_how_often_eat_doughnuts_sweetrolls_danish_muffins_or_pop_tarts | | | | foo:number_of_times |
|  | foo:during_past_month_how_often_eat_any_kind_of_cheese | | | | foo:number_of_times |
|  | foo:how_often_eat_yogurt | | | | foo:number_of_times |
|  | foo:김_미역_다시마등의_해조류를_섭취합니다 | | | | foo:number_of_days |
|  | foo:how_often_eat_mayonnaise_regular_fat | | | | foo:number_of_times |
|  | foo:brown_rice_1_cup | | | | foo:number_of_times |
| **Competency Question 5. assessing general degree** | | | | | |
| SQWRL syntax | foo:Data_element(?x) ^ foo:Measurement_unit(?u) ^ foo:hasMeasProperty(?x, foo:extent) ^ foo:hasRespUnit(?x, ?u) -> sqwrl:select(?x, ?u) | | | | |
| x, u | foo:억제_할수없이_폭식한_적이_있다 | | | | foo:Likert_degree |
|  | foo:과식을_합니까 | | | | foo:Likert_degree |
|  | foo:곡류_쌀밥_잡곡밥_국수_빵등_를_매끼_먹습니까 | | | | foo:Likert_degree |
|  | foo:long_eating_period_indicator | | | | foo:Likert_degree |
|  | foo:it_take_longer_to_eat | | | | foo:Likert_degree |
| **Competency Question 6. assessing applicability (ie., true or false)** | | | | | |
| SQWRL syntax | foo:Data_element(?x) ^ foo:hasMeasProperty(?x, foo:truth) ^ foo:hasRespUnit(?x, ?u) -> sqwrl:select(?x, ?u) | | | | |
| x, u | foo:우유나_유제품_요구르트_치즈_등을_매일먹는다 | | | | foo:binaryE |
|  | foo:과자_초콜릿_사탕_청량음료등_단음식을_즐겨먹습니다 | | | | foo:binaryE |
|  | foo:단음식_아이스크림_케이크_스낵_탄산음료_꿀_엿_설탕등을_매일_섭취한다 | | | | foo:binaryE |
|  | foo:단음식_아이스크림_케이크_스낵_탄산음료_꿀_엿_설탕등을_매일_섭취한다 | | | | foo:binary |
|  | foo:계란노른자_어육류의내장_간_곱창_오징어등을_자주_드십니까 | | | | foo:binary |
|  | foo:지방이_많은_육류_삼겹살_갈비등_는3_일에_1회이상_먹는다 | | | | foo:binaryE |
|  | foo:eat_till_uncomfortably_full | | | | foo:binary |
|  | foo:과일을_매일_먹는다 | | | | foo:binaryE |
|  | foo:햄_라면_햄버거_인스턴트_가공식품_섭취 | | | | foo:binaryE |
|  | foo:과식하지_않는다 | | | | foo:binaryE |
|  | foo:단음식_과자_초콜릿_꿀_아이스크림_청량음료_설탕이_많이_들어있는음식을_많이_드십니까 | | | | foo:binary |
|  | foo:단음식_과자_초콜릿_꿀_아이스크림_청량음료_설탕이_많이_들어있는음식을_많이_드십니까 | | | | foo:binaryE |
|  | foo:매일_잡곡밥 | | | | foo:binaryE |
|  | foo:어패류_생선_오징어_조개등_를_일주일에_3번이상_먹는다 | | | | foo:binaryE |
|  | foo:기름이많은고기_삼겹살_갈비_가공식품_햄_소세지_생크림케이크_버터등을_많이_드십니까 | | | | foo:binary |
|  | foo:육가공식품_햄_베이컨_소시지등이나_라면_인스턴트식품을_1주일에_3회이상_먹는다 | | | | foo:binaryE |
|  | foo:식사속도는_평균_10분_이상이다 | | | | foo:binaryE |
|  | foo:육류나_계란을_일주일에_5번이상_먹는다 | | | | foo:binaryE |
|  | foo:음식에_대한_조절력을_상실하여_한꺼번에_많은양의_음식을_매우_빨리먹는다 | | | | foo:binary |
| **Competency Question 7. assessing the behavior of the past month** | | | | | |
| SQWRL syntax | foo:Data_element(?x) ^ foo:hasObsPeriod(?x, foo:past_month) -> sqwrl:select(?x) | | | | |
| x | foo:during_past_month_how_often_drink_100_fruit_juice_such_as_orange_mango_apple_and_grape_juices | | | | |
|  | foo:during_past_month_how_often_eat_whole_grain_bread_including_toast_rolls_and_in_sandwiches | | | | |
|  | foo:during_past_month_how_often_eat_doughnuts_sweetrolls_danish_muffins_or_pop_tarts | | | | |
|  | foo:during_past_month_how_often_eat_any_kind_of_cheese | | | | |
|  | foo:during_past_month_how_often_eat_fench_fries_home_fries_or_hash_brown_potatoes | | | | |
|  | foo:during_past_month_how_often_eat_cookies_cake_pie_or_brownies | | | | |
|  | foo:during_past_month_how_often_eat_cooked_dried_beans_such_as_refried_beans_baked_beans_bean_soup_and_port_and_beans | | | | |
|  | foo:during_past_month_how_often_eat_fruit | | | | |
| **Competency Question 8. assessing the behavior of the past 6 months** | | | | | |
| SQWRL syntax | foo:Data_element(?x) ^ foo:hasObsPeriod(?x, foo:past_6_months) -> sqwrl:select(?x) | | | | |
| x | foo:how_many_days_per_week_on_average_over_the_past_6_months_you_eat_an_usually_large_amount_of_food_and_experienced_a_loss_of_control | | | | |
| **Competency Question 9. questions on the frequency of high calorie food intake** | | | | | |
| SQWRL syntax | foo:Data_element(?x) ^ foo:High_calorie_food(?h) ^ foo:hasFoodExample(?x, ?h) ^ foo:hasMeasProperty(?x, foo:frequency) -> sqwrl:select(?x) | | | | |
| x | foo:how_often_eat_french_fries_home_fries_or_hash_brown_potatoes | | | | |
|  | foo:how_often_eat_macaroni_and_cheese | | | | |
|  | foo:how_often_eat_deep_fried_foods_away_from_home_or_as_take_out_eg_french_fries_fried_chicken_fish_calms_shrimp_etc | | | | |
|  | foo:during_past_month_how_often_eat_doughnuts_sweetrolls_danish_muffins_or_pop_tarts | | | | |
|  | foo:during_past_month_how_often_eat_cookies_cake_pie_or_brownies | | | | |
|  | foo:how_often_eat_pizza | | | | |
|  | foo:during_past_month_how_often_eat_french_fries_home_fries_or_hash_brown_potatoes | | | | |
| **Competency Question 10. questions on the applicability of unhealthy eating behavior** | | | | | |
| SQWRL syntax | foo:Data_element(?x) ^ autogen0:Unhealthy_dietary_behavior(?u) ^ foo:hasConceptDomain(?x, ?u) ^ foo:hasMeasProperty(?x, foo:truth) ^ foo:hasRespUnit(?x, ?r) -> sqwrl:select(?x, ?u, ?r) | | | | |
| x, u, r | foo:음식에_대한_조절력을_상실하여_한꺼번에_많은양의_음식을_매우_빨리먹는다 | | foo:eating_fast | foo:binary | |
|  | foo:음식에_대한_조절력을_상실하여_한꺼번에_많은양의_음식을_매우_빨리먹는다 | | foo:binge_eating | foo:binary | |
|  | foo:육가공식품_햄_베이컨_소시지등이나_라면_인스턴트식품을_1주일에_3회이상_먹는다 | | foo:processed_meat_intake | foo:binaryE | |
|  | foo:육가공식품_햄_베이컨_소시지등이나_라면_인스턴트식품을_1주일에_3회이상_먹는다 | | foo:instant_food_intake | foo:binaryE | |
|  | foo:과식하지_않는다 | | foo:overeating | foo:binaryE | |
|  | foo:햄_라면_햄버거_인스턴트_가공식품_섭취 | | foo:processed_food_intake | foo:binaryE | |
|  | foo:햄_라면_햄버거_인스턴트_가공식품_섭취 | | foo:instant_food_intake | foo:binaryE | |
|  | foo:eat_till_uncomfortably_full | | foo:overeating | foo:binary | |
